# Influence of enhancement filters in apical bone loss measurement: A cone-beam computed tomography study

**DOI:** 10.4317/jced.53496

**Published:** 2017-04-01

**Authors:** Emerson-Tavares de Sousa, Mayara-Abreu Pinheiro, Patrícia-Pereira Maciel, Marcelo-Augusto-Oliveira Sales

**Affiliations:** 1 DDS, MS. Ph.D. Student. Faculty of Dentistry at Piracicaba – Campinas University, Piracicaba-Brazil; 2 DDS, MS. School of Dentistry, Federal University of Paraiba, Joao Pessoa-Brazil; 3 DDS, MSc, Ph.D. Department of Clinics and Social Dentistry, School of Dentistry, Federal University of Paraiba, Joao Pessoa-Brazil

## Abstract

**Background:**

The use of cone-beam computed tomography images (CBCT) providing a better assessment of bone injuries, although the sensibility of lesions measurement might be improved by the use of enhancement filters. Objective: This study aimed to analyze the influence of enhancement filters in apical bone loss measurement.

**Material and Methods:**

Eighteen CBCT cases randomly selected of apical bone loss were evaluated. The analyses were carried out following the evaluation in axial, coronal and sagittal protocols, using enhancement filters as Hard, Normal, and Very Sharp. The variables were statistically analyzed by Friedman and Wilcoxon test, Spearman’s rho, and intraclass correlation coefficient.

**Results:**

The differences between filters in axial and sagittal protocols were significant (*p*<0.05); however, this was not observed in the coronal slice. The use of Hard filter demonstrates better results than Very Sharp and Normal filter, improving significantly the bone loss measurement. A strong, significant and positive correlation was noted for all filters (with *p*< 0.001), such as a strong agreement between the variables, when the Normal filter was used as a reference.

**Conclusions:**

The use of enhancement filters increases the sensitivity of alveolar bone loss measurement, with relative advantage for Hard filter.

** Key words:**Cone-Beam computed tomography. endodontics. periapical periodontitis. image enhancement. alveolar bone loss.

## Introduction

The endodontic treatment suggests a clinical and radiographic monitoring assess persistent apical lesions and bone loss. In this context, the use of x-ray images provides a decisive role for follow-up the progression of lesions and control the integrity of the tooth and periodontal structures (1).

The radiographic examinations were considered essential for the diagnosis of alveolar bone loss; however, this method has some limitations such as two-dimensionality and impossibility of accurate measurements (2). Therefore, the cone-beam computed tomography (CBCT) images contributes for the better visualization and measurement of structures in three anatomical planes (axial, sagittal and coronal), improving professionals diagnose capacity, decision-making, and elaboration of the treatment plan (3,4).

Currently, the CBCT is the gold standard for root canals morphology visualization and diagnosis injuries as fractures and resorption (5,6). The advantage of this method is the possibility of volumetric data by 3-dimensional reconstruction (7) and acquisition of images with graphical accuracy, compatible with real measurements (4). However, the analysis of images without computational pretreatment might not be the better option for osteolytic lesion diagnosis, considering the visual accuracy of the clinicians. As a result, the enhancement filters have been used such as a computational tool that provides a contrast modification surrounding the lesion, improving the professional sensitivity and specificity involved in the diagnosis, justifying their use in independent workstations (8-10).

Several studies have evaluated the effect of enhancement filters on root fracture and caries (11-13); however, few studies have assessed this effect in alveolar bone loss (8). Thus, this study aimed to analyze the influence of enhancement filters in apical bone loss measurement, using a cone-beam computed tomography in different observation protocols.

## Material and Methods

This is a retrospective study that used 484 images obtained from patients of a private clinic. The images initially selected according to the presence of apical bone loss, and after a computation procedure of randomization by SPSS for Windows (SPSS Inc., v21.0, Chicago, IL, USA) software, 18 cases were included in this investigation. The same radiology technologist took the scans, following a standardized protocol for positioning and exposure. This study was approved by a Brazilian Ethics Committee (169/10 CEP/HULW) that fulfill all the ethical principles of the Helsinki Declaration.

The images were acquired by i-CAT® Cone-Beam 3D Dental Imaging System (Imaging Sciences International, Hatfield, PA, USA) using default parameters (120 kVp, 23.87 mAs, 6 cm field of view, 0.25 mm voxel size, 40s scan time, high-resolution bone filter). The DICOM data obtained were analyzed with a software program (i-CAT Vision TM Vision Q version 1.8.1.10).

In a pilot study, a radiologist with experience in CBCT, trained during one year, one dentistry student for use and measurement in i-CAT® Vision software. The experimental phase was performed sequentially and blindly, in a controlled environment, without light and external stimuli. The images were analyzed beginning by the Normal filter at the point with the biggest area of the lesion, and in sequence, the enhancement filters were used Hard and Very Sharp. This protocol following the usage of the axial, coronal and sagittal slices. The dynamic evaluation was performed using all slices and the zoom tool.

The software program used for statistical analysis was SPSS for Windows version 21. Data normality was obtained by Shapiro-Wilk test. The statistical inference was carried out using Friedman and Wilcoxon test, Spearman’s rho (ρ) and intraclass corre-lation coefficient (ICC). A significance level of 5% (*p* <0.05) was adopted for the two-toiled test.

## Results

The axial protocol showed significant differences in medians on three enhancement filters (*p* = 0.05). The statistical difference between pairwise comparisons was adequately controls for the type I error, that demonstrate significance at 0.05 alpha level considering the comparisons Normal-Hard and Hard-Very Sharp, with better results for Hard filter. The sagittal protocol showed significant differences between filters (*p* = 0.02). The Hard filter had higher measures than the Normal filter (*p*<0.05), however, the difference between Very Sharp-Normal and Very Sharp-Hard filters was not identified (*p*> 0.05). No difference was observed in coronal protocols ( Table 1).

**Table 1:**
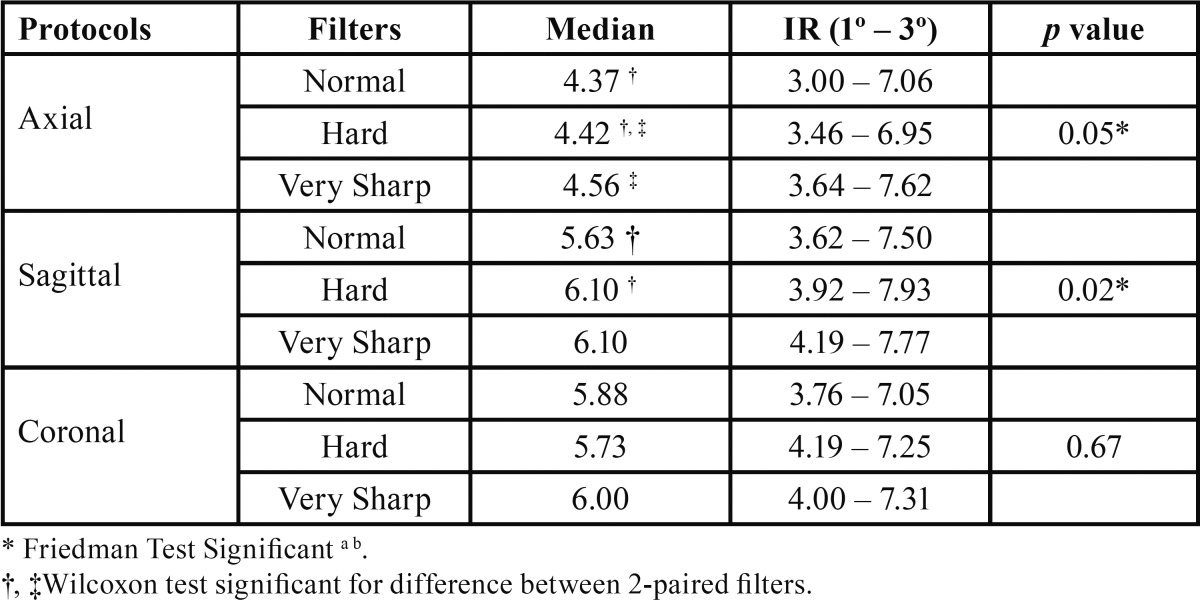
Evaluation of enhancement filters to detecting apical bone loss in different protocols.

Spearman’s correlation (ρ) pointed strong, positive and significant correlation in all filters. The ICC was also calculated to verify the agreement between the filters, ranging 0.97 - 0.99, which suggests a strong correlation between filters, as well as their validity ( Table 2).

**Table 2:**
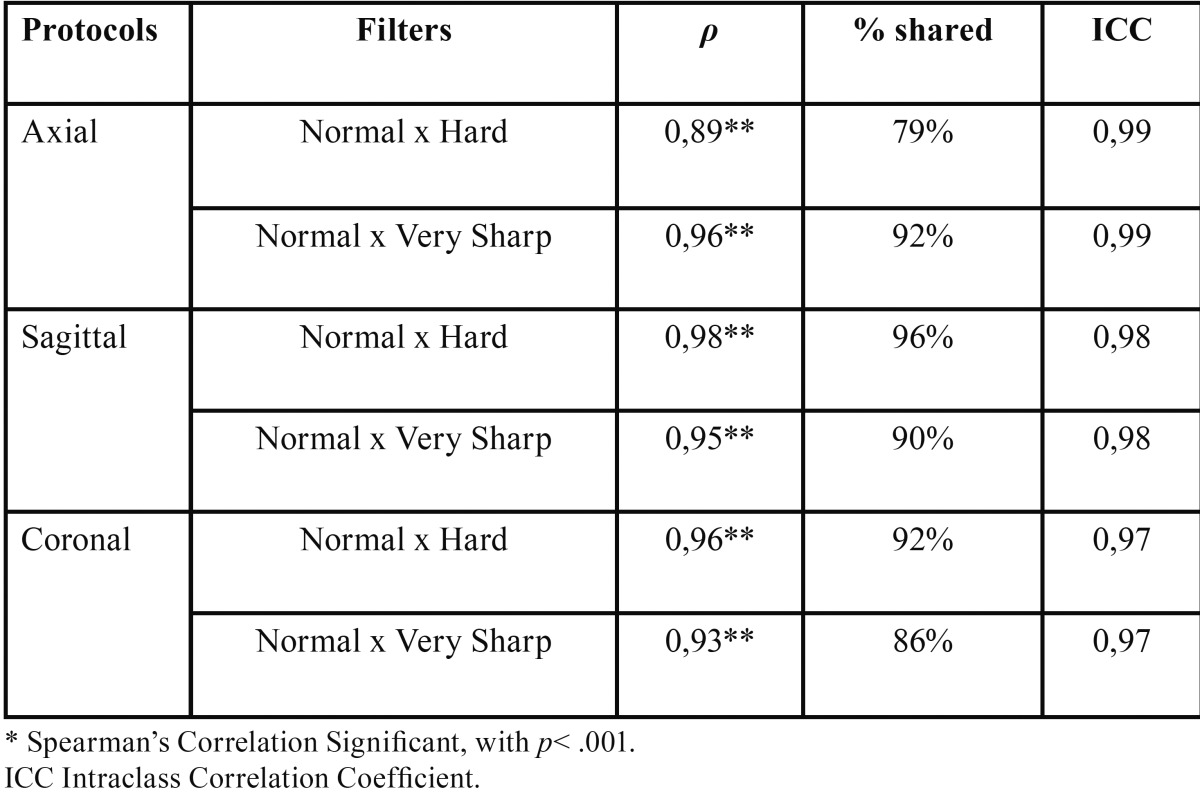
Correlation and Agreement between enhancement filters to detecting apical bone loss in different protocols.

## Discussion

This study found that the enhancement filters on CBCT images influencing the apical bone loss measurement, increasing the sensibility of diagnosis in some different observation slices. The images were analyzed using the methodology established by Monteiro *et al.* (8), who studied the identification of mandible osteolytic lesions that easily might be extrapolated for this research.

It was observed that the visualization of the bone loss might be different when filters are used, what can be explained by the modification of the visualization’s perspective without altering the quality of images. These findings demonstrate that the protocols of visualization influenced the exams interpretation, corroborating by Schulze *et al.* (14) and Monteiro *et al.* (8).

The Hard filter was the most sensitive enhancement tool for detection and measurement of lesions, presenting the highest values when compared with both Normal and Very Sharp filter. In the sagittal protocol, the difference between Very Sharp and Normal filter cannot be observed. However, the use of “Hard” or “Very Sharp” filters has a valid application, considering the significant strong positive correlation when compared with normal filter, without improvement. This finds suggests, subjectively and objectively, that the enhancement filter makes the analysis more sensitive for increasing the image contrast, facilitating the visualization of bone loss areas. The study of Monteiro *et al.* (8) concluded that Normal filter has the lower power of visualization of osteolytic lesions in jaws.

Similarly, the influence of enhancement filters in CBCT diagnosis has been evaluated in several studies to improve the detection of peri-implant dehiscence (15), vertical root fractures (13,16) and mandibular osteolytic lesions (8). Suomalainen *et al.* (17) found that the use of post-processing filters allowed better visualization of bony structures. Thus, to make a visible diagnostic information for the human eye, enhancement filters have been used to manipulate images, improving aspects originally not apparent (18).

Additionally, in all the slices analyzed the variation in Normal filter might also occur to Hard and Very Sharp filter ( Table 2) that indicates a reproducibility of the tested filters and the magnitude of the correlation (*p* > 0.89, *p*< 0.001). These results emphasize that the measures of alveolar bone loss by different filters are reproducible, but the difference of sensibility of lesion size might be different when more than one filter are used to control the progression of the lesion. Thus, we recommend the use of the same filter throughout the review process, to prevent any change, even small, that might influence the follow-up and the decision-making process.

According to Monteiro *et al.* (8), filters algorithms influence the CBCT images, improving CT scans visualization, with the best results confirmed for Very Sharp filter, explaining its clinical use, followed by Hard filters and Normal. In contrast, our results demonstrated that the advantage of Very Sharp filter was not evident, although the results agreement that the use of enhanced filters shows excellent values and demonstrate a valid method with a clinical application for apical bone loss measurements.

This study had some limitations that emerge from the specific nature of radiologic evaluations by CBCT and operational difficulties. First, the absence of a gold standard of evaluations that should be provided by the use of dry skulls. Second, although the reproducibility of the measurements and the controlled assessment by one researcher, the evaluation criteria was always subjective. Third, the study design did not provide an assessment of the lesion progression across the time, what might be a great possibility for other studies.

## Conclusions

The Hard filter used in CBCT images influence the apical bone loss measurement. This research also indicates that the measures of alveolar bone loss are reproducible, but different when the protocol of evaluation is modified. This find suggests that for accurate measurement of alveolar bone loss, the clinician has to standardize the CBCT analyzes, avoiding differences in changing filters that contribute for improper follow-up.

## References

[1] Okada K, Rysavy S, Flores A, Linguraru MG (2015). Noninvasive differential diagnosis of dental periapical lesions in cone-beam CT scans. Med Phys.

[2] Yoshioka T, Kikuchi I, Adorno CG, Suda H (2011). Periapical bone defects of root filled teeth with persistent lesions evaluated by cone-beam computed tomography. Int Endod J.

[3] Scarfe WC, Li Z, Aboelmaaty W, Scott SA, Farman AG (2012). Maxillofacial cone beam computed tomography: essence, elements, and steps to interpretation. Aust Dent J.

[4] Tyndall DA, Kohltfarber H (2012). Application of cone beam volumetric tomography in Endodontics. Aust Dent J.

[5] Durack C, Patel S, Davies J, Wilson R, Mannocci F (2011). Diagnostic accuracy of small volume cone beam computed tomography and intraoral periapical radiography for the detection of simulated external inflammatory root resorption. Int Endod J.

[6] Ponder SN, Benavides E, Kapila S, Hatchd NE (2013). Quantification of external root resorption by low- vs high-resolution cone-beam computed tomography and periapical radiography: A volumetric and linear analysis. Am J Orthod Dentofacial Orthop.

[7] Corbella S, Fabbro MD, Tamse A, Rosen E, Tsesis I (2014). Cone beam computed tomography for the diagnosis of vertical root fractures: a systematic review of the literature and meta-analysis. Oral Surg Oral Med Oral Pathol Oral Radiol.

[8] Monteiro BM, Nobrega Filho DS, Lopes PML, Sales MAO (2012). Impact of Image Filters and Observations Parameters in CBCT for Identification of Mandibular Osteolytic Lesions. Int J Dent.

[9] Hassan B, Van Der Stelt P, Sanderink G (2009). Accuracy of three-dimensional measurements obtained from cone beam computed tomography surface-rendered images for cephalometric analysis: influence of patient scanning position. Eur J Orthod.

[10] Utumi ER, Perrella A, Albuquerque MA, Adde CA, Rocha RG, Cavalcanti MGP (2009). Evaluation of simulated bone lesion in the head of the mandible by using multislice computed tomography. J Appl Oral Sci.

[11] Haiter-Neto F, dos Anjos Pontual A, Frydenberg M, Wenzel A (2008). Detection of non-cavitated approximal caries lesions in digital images from seven solid-state receptors with particular focus on task-specific enhancement filters. An ex vivo study in human teeth. Clin Oral Investig.

[12] Kamburoglu K, Murat S, Pehlivan SY (2010). The effects of digital image enhancement on the detection of vertical root fracture. Dent Traumatol.

[13] Nascimento MC1, Nejaim Y, de Almeida SM, Bóscolo FN, Haiter-Neto F, Sobrinho LC (2014). Influence of cone beam CT enhancement filters on diagnosis ability of longitudinal root fractures. Dentomaxillofac Radiol.

[14] Schulze D, Blessmann M, Pohlenz P, Wagner KW, Heiland M (2006). Diagnostic criteria for the detection of mandibular osteomyelitis using cone-beam computed tomography. Dentomaxillofac Radiol.

[15] Ferreira LM, Visconti MAPG, Nascimento HA, Dallemolle RR, Ambrosano GM, Freitas DQ (2015). Influence of CBCT enhancement filters on diagnosis of vertical root fractures: a simulation study in endodontically treated teeth with and without intracanal posts. Dentomaxillofac Radiol.

[16] Azevedo-Vaz SL, Alencar PNB, Rovaris K, Campos PSF, Haiter-Neto F (2013). Enhancement cone beam computed tomography filters improve in vitro periimplant dehiscence detection. Oral Surg Oral Med Oral Pathol Oral Radiol.

[17] Suomalainen A, Vehmas T, Kortesniemi M, Robinson S, Peltola J (2008). Accuracy of linear measurements using dental cone beam and conventional multislice computed tomography. Dentomaxillofac Radiol.

[18] Borg E, Gröndahl K, Persson LG, Gröndahl HG (2000). Marginal bone level around implants assessed in digital and film radiographs: in vivo study in the dog. Clin Implant Dent Relat Res.

